# Antral follicle size in the downregulated cycle and its relation to *in vitro* fertilization outcome

**DOI:** 10.4103/0974-1208.57225

**Published:** 2009

**Authors:** Nabaneeta Padhy, M Latha, B Sathya, Thangam R Varma

**Affiliations:** Department of Reproductive Medicine, Institute of Reproductive Medicine, Madras Medical Mission, Chennai - 37, India

**Keywords:** Antral follicle size, folliculogenesis, quality of oocytes, zooming

## Abstract

**AIM:**

In this study, we have attempted to relate the antral follicle size on day 3 of downregulation to the *in vitro* fertilization (IVF) outcome and hence test its predictive value for IVF outcome.

**SETTINGS AND DESIGN:**

Teaching hospital, prospective double-blinded cohort study. The sonographer was blinded toward the patient profile whereas the follicular size on day 3 was concealed from the clinicians.

**MATERIALS AND METHODS:**

Two hundred and twenty-eight patients undergoing the long protocol programme for IVF/intracytoplasmic sperm injection at the Institution are included in this study. The antral follicle size on day 3 of the downregulated cycle was measured for all patients and, based on the size, they were divided into three groups: Group I (-3 mm), Group II (3-6 mm), and Group III (6-9 mm), Various outcome measures taken into account were amount and number of days of gonadotropin required, basal estradiol and follicle-stimulating hormone (FSH) level, zooming of follicles, and quality of oocytes.

**STATISTICAL ANALYSIS USED:**

Data were analyzed using the Graphpad software with a microsoft excel spread sheet. A *P*-value < 0.05 (Fisher exact test) was taken to be significant. Multinomial regression tests were used as appropriate.

**RESULTS:**

A significant number of follicles were in the 3-6 mm group whereas the population below 35 years constituted the majority. There was no significant difference in basal estradiol and FSH levels among the three groups. Accelerated growth of follicles (zooming) was significantly associated with bigger antral follicles (*P* < 0.001) whereas poor quality oocytes were significantly higher in Group 1.

**CONCLUSION:**

The significant number of poor quality of oocytes produced by such follicles whereas zooming of follicles among the bigger antral follicle group suggest their accelerated development potential and hence the dose of gonadotropin should be adjusted accordingly, indicating evidence of intrinsic abnormality of folliculogenesis in very small follicles.

## INTRODUCTION

There has been a continuous effort to provide an optimum environment for a successful controlled ovarian hyperstimulation (COH) programme. Various fields have been explored for this and during recent years there is increased interest in understanding the mechanism that regulates follicular growth during early gonadotropin-independent stages of development. Theoretically, every primordial follicle has the potential to mature and ovulate, but this seldom happens.[1] Folliculogenesis is a complex process in which there exists a delicate balance between recruitment and atresia of follicles. Transition from primordial to primary follicular stage does not show any change in the mean diameter of the oocyte, although there is an increase in the follicular diameter, indicating that this transition is a slow maturing rather than a growing process.[2] Although early folliculogenesis is independent of follicle-stimulating hormone (FSH) due to lack of receptors, FSH is required to protect these follicles from atresia. More so, by a direct or indirect action, pituitary hormones such as prolactin, TSH, and growth hormone act synergistically with luteinizing hormone (LH) and FSH to enhance the entry of non-growing follicles into the growth phase.[3] Hence, early folliculogenesis depends on various autocrine and paracrine mechanisms, which is difficult to interpret accurately. However, the size of the antral follicle does reflect the internal milieu and can predict dynamics of follicular growth. Studies have shown that during the later luteal phase the largest healthy follicles are between 2 and 5 mm in diameter.[4] Keeping this in view, we have made an effort to relate the antral follicular size on day 3 of the downregulated cycle to *in vitro* fertilization (IVF) outcome.

## MATERIALS AND METHODS

Two hundred and twenty-eight patients undergoing the long protocol programme for IVF/intracytoplasmic sperm injection at the Institute of Reproductive Medicine and Women's Health, Madras Medical Mission, are included, wherein eight cycles were cancelled due to poor response. The indications for IVF were unexplained infertility, tubal factor, endometriosis, poor ovarian reserve, and male factor infertility. All the patients were downregulated using Lupride (250 μg- 1 mg, depending on the basal LH) from day 21 of the previous cycle and were given an oral contraceptive pill for withdrawal bleed. Patients having persistent clear cysts were not included whereas those with endometriotic cysts were included in the study. After 10-12 days of gonadotropin-releasing hormone agonist administration, the day 3 follicular size was measured. The size of all antral follicles was measured and the patient was allocated into a group according to the mean follicular size. Based on the size, the patients were divided into three groups: Group I (0-3 mm), Group II (3-6 mm), and Group III (6-9 mm). Follicles larger than 9 mm were not included in the study group. All the patients were scanned using sonoline G 50 2D transvaginal probe of 7.5 MHz (siemens, germany) by a single observer to avoid observer bias. Various parameters like age and gynecological disorders like polycystic ovarian syndrome (PCOS) and endometriosis were correlated with the antral follicle size. Outcome measures taken into account were amount and number of days of gonadotropin required, basal estradiol and FSH level, zooming of follicles, and quality of oocytes. Zooming of follicles was defined as rapid growth in follicular size to reach 14 mm or more on day 6 of gonadotropin therapy or those follicles which were ready by day 8 of gonadotropin therapy. Various morphological criteria were used to define quality of oocytes at par with international standards.

## RESULTS

**Figure 1:**
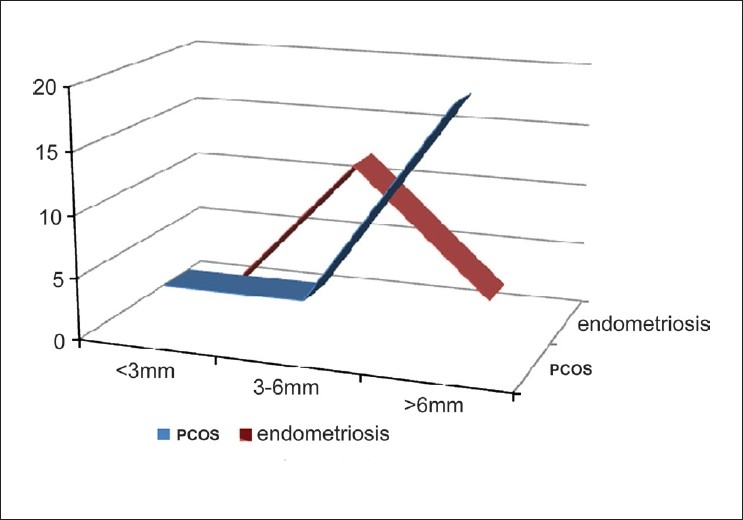
PCOS vs endometriosis – antral follicle size Note: In polycystic ovary syndrome, antral follicles more than 6 mm are abundant whereas in endometriosis, a majority are 3-6 mm in size

Of 220 patients, 176 (80%) were less than 35 years of age and constituted the majority in all three groups, whereas 178 (80.6%) had primary subfertility. Male factor infertility accounted for 26% of the cases [Table 1]. 51.3% of the patients had 3-6 mm follicles, whereas only 18% had follicles less than 3 mm. Very small follicles as well as larger antral follicles were found in younger age groups, which signify that follicle size does not change with age [Table 2]. Majority of PCOS patients had larger antral follicles (*P* < 0.001) whereas in patients with endometriosis, follicle size was not significantly correlated with any group (*P* = 0.06) [Figure 1]. There was no significant difference in basal estradiol and FSH levels among the three groups [Table 3]. Also, there was no difference in the amount of gonadotropin used, as this study was double blinded, and there was a difference in the median days of gonadotropin therapy (Group 1, *P* = 0.02). Accelerated growth of follicles (zooming) was significantly associated with bigger antral follicles (*P* < 0.005) [Table 4]. Poor quality oocytes were significantly high in Group 1.

**Table 1:** Clinical profile of patients

Patient characteristics	n = 220	
	
	<35 years n = 176	>35 years n = 44
Mean age of the patients	28.5	37.9
Duration of infertility (months)	12-45	18-52
Median		
Indication for IVF/		Tubal factor - 25
ICSI		Male factor - 72,
(n = 220)		Unexplained - 26
		Endometriosis - 12
		Poor ovarian reserve - 37
		PCOS- 28
Type of infertility	Primary - 178	Secondary - 42
(n = 220)		

**Table 2:** Antral follicle size in relation to age

Follicle size	<35 years	>35 years	*P*-value
0-3 mm (n = 32)	28	4	0.02
3-6 mm (n = 112)	84	28	0.003
6-9 mm (n = 76)	64	12	0.005

Fisher exact testgonadotropin

**Table 3:** Relationship of basal FSH and estradiol to antral follicle size

Size	Mean E_2_ (mm)	Mean FSH	Mean amount	Days of gonadotropin	GTH (median days)
0-3	11.2 ± 5.34	5.2 ± 1.21	2978 ± 62.41	12-17 (14.2)	*P* = 0.02
3-6	14.9 ± 7.66	5.5 ± 2.43	2675 ± 43.32	9-13 (10.7)	*P* = 0.07
6-9	15.2 ± 6.87	5.0 ± 1.23	2359 ± 21.26	8-12 (9.9)	*P* = 0.08

Fisher exact test using contingency table

Note: As evident from the above table, mean E_2_ and FSH have no variation among the groups and the mean amount of gonadotropin also does not show any significant change. Median days of gonadotropin used is slightly higher in the first group

**Table 4:** Zooming of follicles in relation to antral follicle size n = 220

Follicle size (mm)	Zooming of follicles	*P*-value
0-3	-	
3-6	23/112	0.03
6-9	20/76	0.001

Note: Accelerated development of follicles is significantly higher among bigger follicles whereas no zooming is seen among very small antral follicles

## DISCUSSION

Small antral follicles measuring 2-6 mm have a higher correlation with age.[5,6] However, smaller antral follicles constituted the majority of patients irrespective of age, which probably represents the endocrine reserve in the subfertile group.[5] In contrast, in PCOS patients, there is a cohort of heterogeneous follicles and the bigger antral follicles actually represent the endocrine reserve. Perhaps, the smaller follicles represent the proportion that has undergone premature arrest and hence is associated with follicular arrest in PCOS.[7] However, the developmental potential of oocytes derived from women with PCOS is normal and typically leads to a normal cumulative conception rate.[8,9] Pohl *et al*., in their series of 113 patients, found that patients with a higher number of follicles between 5 and 10 mm showed a significantly higher pregnancy rate.[10]

Zooming of follicles was significantly higher (*P* < 0.001) in Group III patients who had bigger antral follicles. There is emerging evidence for an intrinsic abnormality of folliculogenesis in PCOS that affects the very earliest, gonadotropin-independent stages of follicle development.[11] Although the cause of these early abnormalities are not yet clear, it could be attributed to abnormal serum concentration of FSH, which is suppressed below the threshold level during early follicular phase. Aberrant dynamics of follicle development during early stages leading to disproportionately more granulosa cells per oocyte in transitional and primary follicles may be the cause of such accelerated development.[11] Some authors have also proved that the granulosa cells cultured from follicles derived from anovulatory women with PCOS are hyperresponsive to FSH in terms of estradiol production.[12,13] It is clearly indicated in our study that zooming of follicles is significant high (*P* < 0.001) among Group III patients who had bigger antral follicles.

A significant number of poor quality oocytes (*P* = 0.002) among Group I could be attributed to defective early folliculogenesis [Table 5]. Oocyte quality is determined by the follicular environment rather than by age factors.[14] As the very small antral follicles are not gonadotropin dependent and the number of granulosa cells are less, the response to COH is poor in such follicles and the mean duration of therapy is significantly high in this group.

**Table 5:** Oocyte quality in relation to antral follicle size

Follicle size (mm)	Poor quality oocytes	95% confidence interval	Adjusted
0-3	12/32 (*P* < 0.001)	2.3122-11.9207	5.25
3-6	8/112 (*P* = 0.08)	0.2441-1.8864	0.6786
6-9	8/76 (*P* = 0.056)	0.5301-4.0966	1.5139

OR, Odds ratio; multinomial regression test

Note: Very small antral follicles show a significant number of poor quality oocytes; Optimum response is seen among Group 2 patients

Certainly, patients with follicles <3 mm or >6 mm in the downregulated cycle need more vigilant follow-up and survey. The quality of oocytes is influenced by the internal environment of the follicle, which is reflected in its size. In an era where the COH programme is being optimized in all respects, perhaps our study will add a parameter to predict IVF outcome. But, conclusions could be drawn only after following a larger series of patients.
